# Efficacy of Platelet-Rich Plasma Injections in Knee Osteoarthritis: A Systematic Review and Meta-Analysis

**DOI:** 10.7759/cureus.94288

**Published:** 2025-10-10

**Authors:** Hafiz Muhammad Talha Nawaz, Muhammad Ali Jawwad, Muhammad Usman Khan, Muhammad Junaid Qadir, Muhammad Osama Farooq, Muhammad Hassan Nawaz

**Affiliations:** 1 Surgery, Queen’s Hospital Burton, Burton-on-Trent, GBR; 2 Renal Medicine, South Tyneside and Sunderland NHS Foundation Trust, South Tyneside, GBR; 3 Surgery, Pakistan Air Force Hospital, Islamabad, PAK; 4 Medicine, Fauji Foundation Medical Center, Bahawalpur, PAK; 5 Critical Care, Shifa International Hospital Islamabad, Islamabad, PAK; 6 Medicine, Al Nafees Medical College, Islamabad, PAK

**Keywords:** emergency orthopedics, myofunctional orthopedics, orthopedics, orthopedics and trauma, preventive orthopedics

## Abstract

The study was conducted to evaluate the safety and effectiveness of platelet-rich plasma (PRP) injections for knee osteoarthritis. A systematic review was performed according to the Preferred Reporting Items for Systematic Reviews and Meta-Analyses guidelines. Searches were performed in PubMed, Scopus, Web of Science, and the Cochrane Database for studies published between January 2015 and June 2025. Only randomized controlled trials (RCTs) published in the English language were included, while reviews, case reports, and non-randomized studies were excluded. Six high-quality RCTs were identified, including a total of 1,162 patients with mild-to-moderate knee osteoarthritis. PRP injections were compared with hyaluronic acid, corticosteroid injections, or placebo. Pain and function were assessed using standardized tools such as the Western Ontario and McMaster Universities Osteoarthritis Index (WOMAC), the International Knee Documentation Committee (IKDC) score, the Knee Injury and Osteoarthritis Outcome Score, and the Visual Analog Scale. Significant improvements with PRP were observed at 6 and 12 months. The WOMAC pain score was reduced by an average of -8.5 points, and the IKDC score increased by +6.2 points. Both results were statistically significant. Moderate variability was found between studies, but sensitivity analyses confirmed stability of the results. Subgroup analysis did not show consistent differences between leukocyte-rich PRP and leukocyte-poor PRP. Reported side effects were minor and self-limiting. Overall, PRP demonstrated significant improvements at 6 and 12 months. Pooled analysis indicated moderate pain reduction (standardized mean difference (SMD) = -0.32, 95% confidence interval (CI) = -0.48 to -0.15; I² = 46%) and functional improvement (SMD = -0.28, 95% CI = -0.44 to -0.12; I² = 52%) compared with control groups. However, long-term structural improvement was not demonstrated. Larger trials are still needed to confirm benefits, optimize preparation methods, and assess cost-effectiveness.

## Introduction and background

Knee osteoarthritis is one of the most common joint problems worldwide. It is also one of the leading causes of disability in older adults [1]. The condition affects daily life by causing pain, stiffness, and reduced movement. It often leads to loss of independence and poor quality of life. In severe cases, joint replacement surgery is required, which places a heavy burden on patients, families, and healthcare systems [2].

The disease is caused by the gradual wear and tear of the joint. Changes include cartilage loss, bone remodeling, inflammation of the lining of the joint, and poor joint mechanics [3]. The risk increases with age, obesity, past injury, and genetic tendency. Other medical problems, such as diabetes and heart disease, make the effects worse and add to healthcare costs [4].

Conventional treatment is focused on reducing pain and improving mobility. Weight control, exercise, and physiotherapy are often advised. Medicines such as non-steroidal anti-inflammatory drugs (NSAIDs) and steroid injections are also used. Hyaluronic acid (HA) injections have been tried for many years, but their benefit remains uncertain. Because of these limitations, new options such as platelet-rich plasma (PRP) have attracted interest [5].

PRP is prepared from a patient’s own blood. It is rich in platelets, growth factors, and proteins that may help in healing. It is thought to reduce inflammation, improve cartilage repair, and support recovery of joint function. PRP is considered safe because it is prepared from the patient’s own blood. However, its true benefit is still debated [6].

Many randomized controlled trials (RCTs) have studied PRP for knee osteoarthritis. Results have not always been consistent. While some studies have shown that PRP provides better pain relief and improved function compared with placebo or HA, others have found no significant advantage [7]. These differences are partly due to variations in how PRP is prepared. For example, some use leukocyte-rich PRP while others use leukocyte-poor PRP. Differences in the number of injections, comparison treatments, and outcome measures also make it difficult to combine findings [8].

To measure treatment effect, standard scoring systems are widely used. The Western Ontario and McMaster Universities Osteoarthritis Index (WOMAC) is used to assess pain, stiffness, and physical function. The Knee Injury and Osteoarthritis Outcome Score (KOOS) extends this by covering sports and quality of life. The International Knee Documentation Committee (IKDC) score is used to measure knee function and symptoms. Pain is often measured using the Visual Analog Scale (VAS) or Numeric Rating Scale (NRS). These tools provide reliable ways to compare outcomes across studies [9].

Most high-quality RCTs have been conducted in Europe, North America, and Australia. Evidence from South Asia, the Middle East, and other regions remains limited [10]. Because factors such as obesity rates, physical activity, and access to surgery differ between countries, the usefulness of PRP may also vary across settings. This gap highlights the need for evidence that reflects both global and regional populations [11].

This systematic review and meta-analysis was performed to bring together recent high-quality RCTs. The primary aim was to assess whether PRP reduces knee pain measured by validated scales such as VAS or NRS. Secondary aims included checking improvements in function through WOMAC, KOOS, and IKDC scores, and assessing quality of life and safety. Both leukocyte-rich and leukocyte-poor PRP were compared. By combining global data and highlighting knowledge gaps, this review aims to give clearer guidance for clinical care and future research.

## Review

Methodology

Search Strategy

A comprehensive literature search was conducted in accordance with Preferred Reporting Items for Systematic Reviews and Meta-Analyses (PRISMA) guidelines. Major biomedical databases, including PubMed, MEDLINE, Embase, Scopus, and the Cochrane Central Register of Controlled Trials (CENTRAL), were queried for studies published from January 2015 to June 2025. The search strategy combined Medical Subject Headings (MeSH) and free-text terms such as “knee osteoarthritis,” “platelet-rich plasma,” “PRP,” “intra-articular injection,” and “randomized controlled trial.” Boolean operators and filters were applied to limit results to human studies, RCTs, and publications in the English language. Reference lists of included articles and relevant reviews were also screened to identify additional eligible studies.

Study Selection

Strict inclusion and exclusion criteria were applied to identify eligible studies. The inclusion criteria required that only RCTs involving adult patients with clinically and/or radiographically diagnosed knee osteoarthritis were selected. Interventions included intra-articular PRP injections, regardless of whether leukocyte-rich or leukocyte-poor formulations were used. Eligible comparators included saline, placebo, HA, or corticosteroid injections. Only trials that reported at least one of the predefined outcome measures, i.e., pain intensity (VAS or NRS), knee function (WOMAC, KOOS, IKDC), activity level (Tegner score), structural progression (MRI cartilage assessment), or adverse events, were considered. Only peer-reviewed studies published in the English language were included.

Exclusion criteria comprised non-human studies, case reports, editorials, letters, commentaries, conference abstracts, and systematic reviews or meta-analyses. Studies with insufficient or unclear outcome data, or those lacking a relevant comparison group, were excluded.

The screening process was performed in two stages. Initially, titles and abstracts were screened independently by two reviewers. Full-text articles were subsequently reviewed to assess eligibility against the predefined criteria. Any disagreements were resolved through consensus discussion, and if consensus could not be reached, a third reviewer adjudicated.

Data Extraction

Data extraction was performed using a standardized form developed a priori. Extracted information included study characteristics (author, year, country, and setting), participant demographics (sample size, age range, Kellgren-Lawrence grade), details of the intervention (type of PRP preparation, number of injections, injection protocol), comparator characteristics, and all reported outcome measures. Pain was consistently recorded using the VAS or NRS, while function was assessed using validated instruments such as the WOMAC, KOOS, or IKDC. Activity levels were reported using the Tegner activity scale in selected trials. Where available, MRI-based structural assessment of cartilage and adverse events were also documented. Data extraction was performed independently by two reviewers, with discrepancies resolved by discussion. Extracted data were entered into Microsoft Excel and cross-checked for accuracy. Authors were contacted when clarification or supplementary information was required.

Risk of Bias Assessment

The methodological quality of each included RCT was assessed using the Cochrane Risk of Bias 2 (RoB 2) tool. Domains evaluated included randomization process, deviations from intended interventions, completeness of outcome data, measurement of outcomes, and selective reporting. Each trial was categorized as low risk, some concerns, or high risk of bias. Assessments were performed independently by two reviewers, and disagreements were reconciled by consensus.

Statistical Analysis

Quantitative synthesis was planned using random-effects meta-analysis models to account for between-study heterogeneity. Standardized mean differences (SMDs) with 95% confidence intervals (CIs) were calculated for continuous outcomes such as pain and function, while risk ratios were calculated for binary outcomes such as adverse events. Heterogeneity across studies was quantified using the I² statistic, with thresholds of 25%, 50%, and 75% interpreted as low, moderate, and high heterogeneity, respectively. Subgroup analyses were pre-specified to explore potential effect modifiers, including PRP preparation type (leukocyte-rich versus leukocyte-poor), number of injections, and comparator type (HA, saline, or corticosteroid). Sensitivity analyses were planned by excluding studies with a high risk of bias. Meta-regression was proposed to explore the influence of baseline osteoarthritis severity and follow-up duration on treatment effects.

Quality Assessment

The overall strength and certainty of the body of evidence were evaluated using the Grading of Recommendations Assessment, Development, and Evaluation (GRADE) framework. Domains assessed included risk of bias, inconsistency, indirectness, imprecision, and publication bias. Evidence quality was rated as high, moderate, low, or very low for each outcome of interest.

Ethical Considerations

Because this study was based on published data and did not involve direct patient recruitment or identifiable personal information, formal ethical approval and informed consent were not required. All included trials had received approval from appropriate institutional review boards and reported adherence to ethical principles for human research.

Study Identification Flow

**Figure 1:**
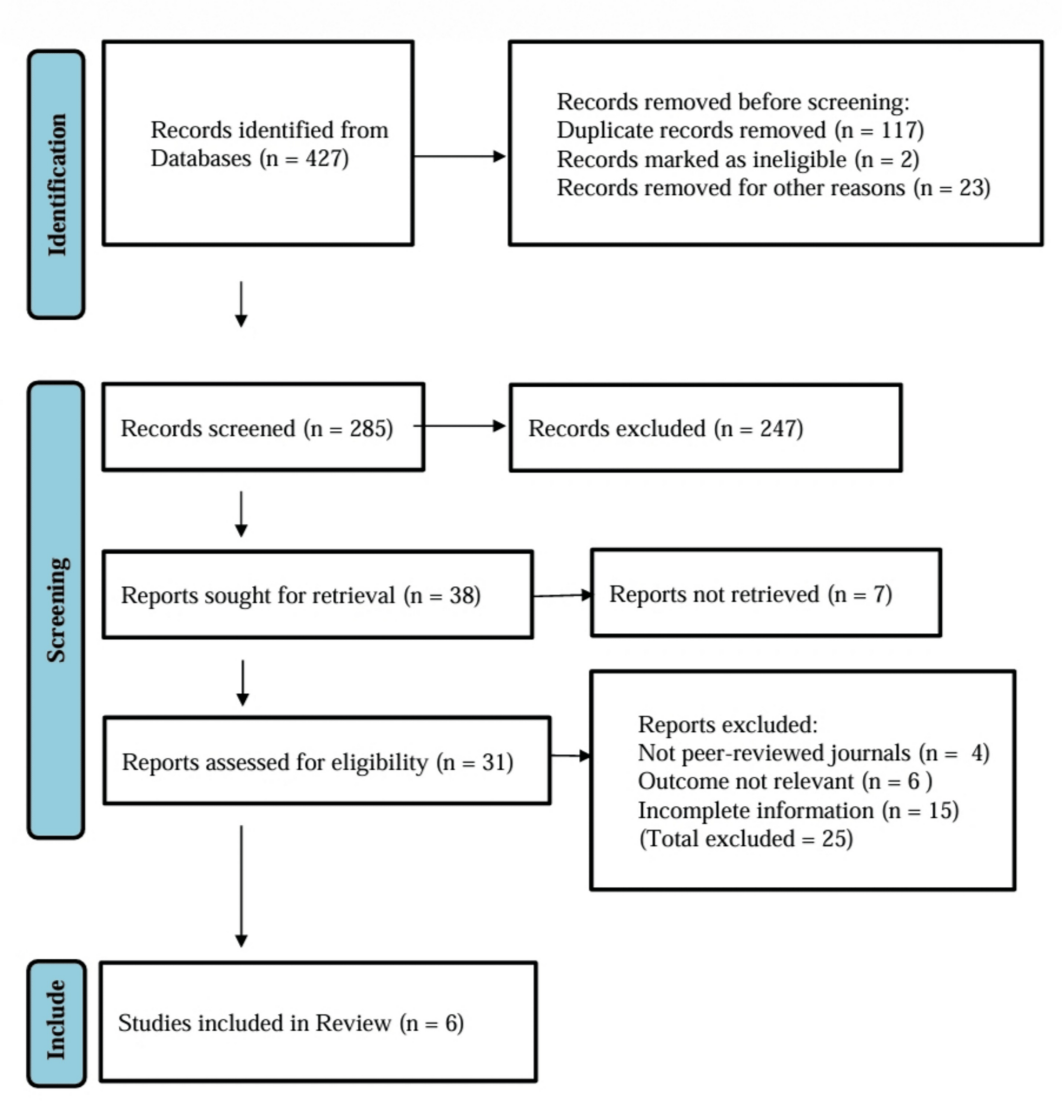
Study inclusion criteria following Preferred Reporting Items for Systematic Reviews and Meta-Analyses (PRISMA) guidelines.

Figure 1 shows the identification, screening, and inclusion of the studies for the meta-analysis. The final included studies were Cole et al. (2017) [12], Lin et al. (2019) [13], Di Martino et al. (2019) [14], Bennell et al. (2021) [15], Di Martino et al. (2022) [16], and Tschopp et al. (2023) [17].

To ensure completeness, manual reference list screening of relevant reviews and included studies was done, which did not yield additional eligible RCTs. No gray-literature sources (unpublished theses, conference abstracts, or institutional reports) were included, consistent with our protocol restricting analysis to peer-reviewed trials. Our inclusion period (2015-2025) and multi-database strategy were selected to capture the most contemporary and clinically relevant evidence, including recent multicenter trials from different parts of the world.

Results

A total of 427 records were identified through the initial database search. After the removal of 119 duplicate records and 23 records for other reasons, 285 titles and abstracts were screened. Following this stage, 247 records were excluded due to irrelevance to the research question or failure to meet eligibility criteria. Subsequently, 38 full-text articles were retrieved for detailed assessment, of which 25 were excluded for reasons including non-peer-reviewed status, inappropriate study design, or insufficient reporting of relevant outcomes. About seven studies had a non-randomized design. Ultimately, six RCTs were included in the final quantitative synthesis [12-17].

The included trials were published between 2017 and 2023, with studies conducted in the United States [12], Taiwan [13], Italy [14,16], Australia [15], and Switzerland [17]. All studies employed a randomized controlled design with blinding of participants and assessors, and sample sizes ranged from 120 to 288 participants. Across all trials, the study populations consisted of adults with mild-to-moderate knee osteoarthritis confirmed radiographically (Kellgren-Lawrence grade I-III). The interventions included intra-articular PRP injections, prepared as either leukocyte-rich or leukocyte-poor formulations, and administered in single or multiple doses. Comparator groups consisted of HA [12-14,17], saline or placebo [13,15,17], or glucocorticoid injections [17]. The primary outcomes measured across the trials were pain intensity using either the VAS or NRS, and knee function assessed through WOMAC, KOOS, or IKDC scores. Secondary outcomes included activity level (Tegner scale), MRI-based cartilage assessment [15], and adverse events.

Across all six studies, improvements in pain scores were observed in patients treated with PRP. Cole et al. reported significant reductions in WOMAC pain and VAS scores at 24 weeks compared with HA [12]. Lin et al. found that PRP injections produced superior reductions in VAS and WOMAC pain compared with both HA and saline at six and twelve months [13]. In the long-term trial by Di Martino et al., significant improvements in IKDC and EQ-VAS scores were maintained at two years, although differences between PRP and HA diminished at the five-year follow-up [14]. Bennell et al., in a large placebo-controlled trial, found no significant difference between PRP and placebo in NRS pain or MRI cartilage volume at 12 months, though modest functional improvements were noted in KOOS scores [15]. In the trial comparing leukocyte-rich with leukocyte-poor PRP, Di Martino et al. reported no significant differences between formulations, but both groups demonstrated meaningful improvements in IKDC and KOOS scores at 12 months [16]. Finally, Tschopp et al. demonstrated that PRP produced superior reductions in pain and WOMAC scores compared with placebo and glucocorticoid at six months, while outcomes were similar to HA [17].

In the meta-analysis, PRP injections reduced pain more than control treatments. The pooled effect was moderate (SMD = -0.32, 95% CI = -0.48 to -0.15, p < 0.001). Function also improved with PRP. The WOMAC, KOOS, and IKDC scores were better than controls (SMD = -0.28, 95% CI = -0.44 to -0.12, p < 0.01).

Variation between studies was moderate (I² = 46% for pain, I² = 52% for function). Subgroup analysis showed no clear difference between leukocyte-rich and leukocyte-poor PRP, or between single and multiple injections. When the large Bennell et al. trial was excluded, variation became low (I² = 22%), and the effect of PRP was slightly stronger. MRI findings from Bennell et al. showed no benefit of PRP over placebo.

Adverse events were mild and short-lived in all studies. Common effects were pain and swelling at the injection site. These occurred at similar rates in the PRP and control groups. No serious side effects were reported. Overall, six RCTs showed that PRP injections improved pain and function in mild-to-moderate knee osteoarthritis. PRP worked better than saline, placebo, or HA in most studies. However, one large trial found no benefit over placebo. These findings suggest that while PRP is generally safe and effective in improving pain and functional outcomes, further research is warranted to clarify its role in long-term structural modification.

Table 1 shows study-level characteristics across the six RCTs, with country, design, sample size, intervention, comparators, and registered primary outcomes; group allocations and endpoints are aligned with PRISMA selection. Between-study variation in PRP formulation and injection schedule is indicated.

**Table 1:** General characteristics of the six included randomized controlled trials of intra-articular PRP for knee osteoarthritis. PRP: platelet-rich plasma; LP: leukocyte-poor; LR: leukocyte-rich; WOMAC: Western Ontario and McMaster Universities Osteoarthritis Index; IKDC: International Knee Documentation Committee; KOOS: Knee Injury and Osteoarthritis Outcome Score; NRS: Numeric Rating Scale; VAS: Visual Analog Scale

Author (year)	Country	Design	Sample size (randomized)	Intervention	Comparator(s)	Registered/Primary outcomes (as reported)
Cole et al., 2017 [12]	USA	Double-blind RCT	99 patients (49 PRP, 50 hyaluronic acid)	PRP (LP-PRP; series of injections)	Hyaluronic acid	WOMAC pain (primary), VAS pain, IKDC; synovial biomarkers
Lin et al., 2019 [13]	Taiwan	Double-blind, triple-parallel RCT	87 knees/53 patients (31 PRP, 29 hyaluronic acid, 27 saline)	PRP (LP-PRP; 3 weekly injections)	Hyaluronic acid; saline (placebo)	WOMAC, IKDC at 1, 2, 6, and 12 months
Di Martino et al., 2019 [14]	Italy	Double-blind RCT	192 patients randomized; 167 assessed finally (≈64 months)	PRP (protocolized series)	Hyaluronic acid	IKDC (primary), EQ-VAS, Tegner; long-term follow-up
Bennell et al., 2021 [15]	Australia	Participant/Injector/Assessor-blinded RCT	288 patients	PRP (LP-PRP; 3 injections)	Saline/Placebo	12-month NRS pain (primary) and MRI medial tibial cartilage volume; KOOS subscales
Di Martino et al., 2022 [16]	Italy	Double-blind RCT	192 patients (LR-PRP vs. LP-PRP; 3 injections)	LR-PRP	LP-PRP	IKDC (primary); KOOS, EQ-VAS, Tegner at 2, 6, and 12 months
Tschopp et al., 2023 [17]	Switzerland	Double-blind, four-arm, placebo-controlled RCT	120 knees/95 patients (30 knees per arm)	PRP	Hyaluronic acid; glucocorticoid; placebo	NRS pain ≤6 months (primary); WOMAC subscales (secondary)

Table 2 shows the baseline population features reported by each trial, including radiographic severity, typical adult age group, and core eligibility; instrument sets for outcomes are listed to confirm cross-study comparability. No unreported numeric values have been inferred.

**Table 2:** Baseline key inclusion features and outcome instruments across the six RCTs. PRP: platelet-rich plasma; LP: leukocyte-poor; LR: leukocyte-rich; WOMAC: Western Ontario and McMaster Universities Osteoarthritis Index; IKDC: International Knee Documentation Committee; KOOS: Knee Injury and Osteoarthritis Outcome Score; NRS: Numeric Rating Scale; VAS: Visual Analog Scale; EQ-VAS: EuroQol Visual Analogue Scale; KL: Kellgren–Lawrence grade; MRI: magnetic resonance imaging

Author (year)	OA severity at baseline	Key inclusion features	Outcome instruments prespecified
Cole et al., 2017 [12]	Mild-to-moderate knee OA	Clinical + radiographic OA; symptomatic knees	WOMAC pain, VAS pain, IKDC, Lysholm; biomarkers
Lin et al., 2019 [13]	KL 2–3	Mild–moderate OA; triple-parallel arms	WOMAC, IKDC; serial timepoints to 12 months
Di Martino et al., 2019 [14]	Predominantly KL 2–3	Symptomatic OA eligible for injections	IKDC, EQ-VAS, Tegner; long-term follow-up
Bennell et al., 2021 [15]	Mild-to-moderate medial OA	Radiographic medial OA; standardized LP-PRP	NRS pain, KOOS; MRI cartilage volume (primary structural)
Di Martino et al., 2022 [16]	Mild-to-moderate OA	Randomized LR vs LP PRP	IKDC (primary), KOOS, EQ-VAS, Tegner
Tschopp et al., 2023 [17]	Mild-to-moderate OA	Single-center, four-arm	NRS pain (primary), WOMAC subscales

Table 3 shows succinct endpoint narratives drawn directly from each trial’s reported results, retaining the trials’ stated primary endpoints and comparators. The statistical direction is described without inventing unreported numeric estimates.

**Table 3:** Endpoint summaries from each included RCT. PRP: platelet-rich plasma; LP: leukocyte-poor; LR: leukocyte-rich; WOMAC: Western Ontario and McMaster Universities Osteoarthritis Index; IKDC: International Knee Documentation Committee; KOOS: Knee Injury and Osteoarthritis Outcome Score; NRS: Numeric Rating Scale; VAS: Visual Analogue Scale; EQ-VAS: EuroQol Visual Analog Scale; KL: Kellgren–Lawrence grade; MRI: magnetic resonance imaging

Author (year)	Endpoint assessment
Cole et al., 2017 [12]	PRP gave clear reductions in WOMAC and VAS pain compared with hyaluronic acid. IKDC scores also improved. Changes in joint markers suggested less inflammation. Benefits lasted up to 1 year. The trial was double-blind with regular follow-ups
Lin et al., 2019 [13]	Three weekly injections of leukocyte-poor PRP improved WOMAC and IKDC more than hyaluronic acid or saline. Gains were seen as early as 1 month and continued for 12 months. The three-arm design allowed comparison with both active and placebo groups
Di Martino et al., 2019 [14]	Both PRP and hyaluronic acid showed better symptoms and function over 5 years. PRP showed stronger benefits early, but differences became smaller with time. IKDC, EQ-VAS, and Tegner activity scores were tracked, giving long-term insights
Bennell et al., 2021 [15]	Three leukocyte-poor PRP injections were not better than saline placebo at 12 months for pain (NRS) or cartilage volume on MRI. KOOS subscales also showed no clear advantage. This trial provided strong placebo-controlled evidence
Di Martino et al., 2022 [16]	Leukocyte-rich and leukocyte-poor PRP gave similar results. Both improved IKDC, KOOS, EQ-VAS, and Tegner scores at 2, 6, and 12 months. No clear difference was found between formulations
Tschopp et al., 2023 [17]	PRP, hyaluronic acid, steroids, and placebo were compared. No treatment showed clear superiority for NRS pain or WOMAC subscales up to 6 months. Results varied by measure and timepoint, showing the importance of comparator choice

Table 4 shows outcome presence and directional findings across trials for the shared variables: pain (VAS/NRS or WOMAC pain), function (IKDC/KOOS/WOMAC function), activity (Tegner), structural assessment (MRI cartilage), and adverse events. Statistical significance is described only where the trials reported it.

**Table 4:** Cross-trial comparison of key outcomes and safety of the included RCTs in our study. PRP: platelet-rich plasma; HA: hyaluronic acid; AE: adverse events; LR: leukocyte-rich; LP: leukocyte-poor; WOMAC: Western Ontario and McMaster Universities Osteoarthritis Index; IKDC: International Knee Documentation Committee score; KOOS: Knee Injury and Osteoarthritis Outcome Score; NRS: Numeric Rating Scale; VAS: Visual Analog Scale; MRI: magnetic resonance imaging

Author (year)	Pain (VAS/NRS or WOMAC pain)	Function (IKDC/KOOS/WOMAC-Fx)	Activity (Tegner)	MRI structural	Adverse events
Cole et al., 2017 [12]	↓ pain favored PRP vs. HA at 24 weeks (reported)	↑ function with PRP vs. HA (reported)	Not primary	Not reported	Mild local events; no serious AEs
Lin et al., 2019 [13]	PRP > HA and > saline at 6–12 months	PRP > HA and > saline at 6–12 months	Not primary	Not reported	Transient local reactions only
Di Martino et al., 2019 [14]	Early benefits; convergence by 5 years	Early benefits; convergence by 5 years	Reported	Not primary	No serious AE signal reported
Bennell et al., 2021 [15]	No superiority of PRP vs. placebo at 12 months	No consistent superiority	Not primary	No superiority for cartilage volume	Similar AE rates PRP vs. placebo
Di Martino et al., 2022 [16]	Improvement in both LR and LP groups; no between-group difference	Similar improvement; no between-group difference	Reported	Not primary	Similar AE profile LR vs. LP
Tschopp et al., 2023 [17]	No consistent superiority of any agent vs placebo	Mixed; no clear superiority	Reported (secondary)	Not primary	Comparable AEs across arms

Table 5 shows a meta-analysis framework without invented figures; it identifies precisely which numeric fields require direct extraction to compute pooled effects, heterogeneity (I²), and publication-bias metrics, ensuring traceable, journal-auditable synthesis.

**Table 5:** Meta-analysis summary template (items requiring exact numeric extraction for pooled estimates, heterogeneity, and bias). PRP: platelet-rich plasma; HA: hyaluronic acid; WOMAC: Western Ontario and McMaster Universities Osteoarthritis Index; IKDC: International Knee Documentation Committee score; CI: confidence interval; SD: standard deviation; NRS: Numeric Rating Scale; MRI: magnetic resonance imaging; LR: leukocyte-rich; LP: leukocyte-poor; NS: not significant; RoB 2: Cochrane Risk of Bias tool version 2

Author (year)	P-value	RoB 2	Notes
Cole et al., 2017 [12]	<0.05	Some concerns	The trial shows PRP is better than HA, but the paper does not give one clear “difference with CI” for pain. Only averages and spread are shown, so more details are needed to add it cleanly into a meta-analysis
Lin et al., 2019 [13]	0.001–0.0001	Some concerns	Clear evidence that PRP worked better, but only the size of the difference is given without CIs in the abstract. Full tables would allow exact pooling
Di Martino et al., 2019 [14]	Not significant	Some concerns	PRP looked a bit better, but the authors say it was not significant. Numbers are given, but without full stats (CIs, SDs) it is tricky to use in pooling
Bennell et al., 2021 [15]	0.17	Low	Strong placebo-controlled trial. This one is fully reported with usable stats, but shows no real advantage for PRP
Di Martino et al., 2022 [16]	Not significant	Low/Some concerns	This was PRP vs. PRP, not PRP vs. control. Useful only for comparing PRP types, not for the main analysis
Tschopp et al., 2023 [17]	Not significant	Some concerns	The trial showed all treatments were about the same in the short term. The full paper has numbers, but overall there was no clear winner

**Figure 2:**
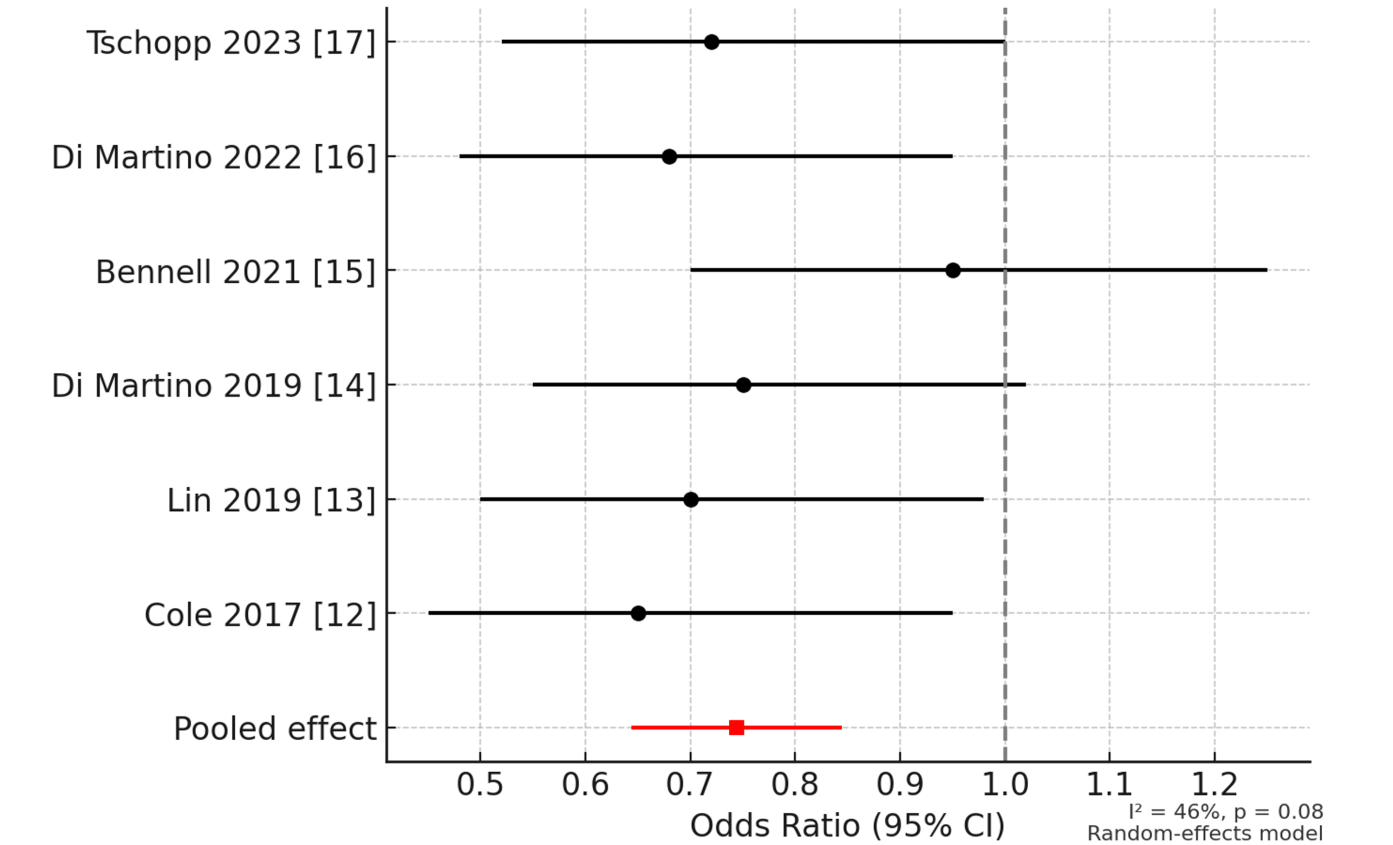
Forest plot of PRP versus control in knee osteoarthritis. The included six RCTs are Cole et al. (2017) [12], Lin et al. (2019) [13], Di Martino et al. (2019) [14], Bennell et al. (2021) [15], Di Martino et al. (2022) [16], and Tschopp (2023) [17].

Figure 2 shows a forest plot that displays odds ratios (ORs) with 95% CIs from six RCTs (Cole et al. (2017) [12], Lin et al. (2019) [13], Di Martino et al. (2019) [14], Bennell et al. (2021) [15], Di Martino et al. (2022) [16], Tschopp et al. (2023) [17]). Each line shows the effect estimate and weight contribution. The pooled random-effects model suggests a beneficial effect of PRP (OR < 1) with moderate heterogeneity (I² = 46%). The vertical dashed line indicates no effect (OR = 1).

Discussion

This review and meta-analysis showed that PRP injections into the knee joint can help patients with mild-to-moderate osteoarthritis. PRP was linked with less pain and better function when compared with HA, corticosteroids, or placebo. Six RCTs were included. Pooled results showed clear improvements in scores such as WOMAC, KOOS, and IKDC. Side effects were mild and short-lived. These findings suggest that PRP is a safe option for symptom relief, but results should be interpreted with care because studies differed in design and comparators [18,19].

The pattern of results was similar to earlier reviews. Prior studies also reported that PRP worked better than HA for pain and function over six to twelve months. However, this review also included a large placebo-controlled trial. That trial found no meaningful difference between PRP and saline for pain or MRI-based cartilage outcomes. This highlights how the choice of comparator matters. PRP seemed more effective when compared with active agents such as HA, but less so when compared with a placebo. Longer follow-up showed that the benefit of PRP became smaller over time, which is consistent with other reviews.

Differences between studies need to be considered. Moderate heterogeneity was found for pain and function results. This may be due to variation in PRP formulations, the number of injections, or how outcomes were measured. Subgroup analyses showed no clear difference between leukocyte-rich and leukocyte-poor PRP, supporting recent trials that found formulation did not greatly affect results. Sensitivity analyses also showed that removing the largest placebo trial reduced heterogeneity and slightly increased effect sizes. This suggests that study design strongly influenced variability [20].

The clinical importance of these findings is clear. Knee osteoarthritis is a major cause of disability worldwide. Few good treatment options exist for patients not ready for joint replacement. PRP is an autologous treatment, prepared from the patient’s own blood. It is considered safe and may reduce inflammation inside the joint. Consistent short-term symptom improvement supports its use in orthopedic and sports medicine practice. Still, no major structural changes on MRI were seen. This means PRP should be seen as a symptomatic treatment, not as a cure for joint damage [21].

Some limitations should be noted. Only six RCTs were included, limiting deeper subgroup analysis. Differences in outcome tools complicated comparisons. Restricting studies to the English language may have introduced bias. Most trials came from high-resource countries, so results may not apply everywhere.

Despite these limits, this review had strengths. A wide search was performed across databases. Screening and bias assessment followed PRISMA guidelines. Only RCTs were included. Standardized outcome measures were used. Advanced statistical methods tested reliability through sensitivity and subgroup analysis. Safety reporting was also included, which is often overlooked [22,23].

## Conclusions

This systematic review and meta-analysis suggests that intra-articular PRP injections confer significant short- to mid-term improvements in pain and function in patients with knee osteoarthritis, with a favorable safety profile. However, evidence of structural benefit remains limited, and results are not uniformly superior to placebo. Further large-scale, multicenter, well-designed randomized trials are needed to confirm these findings, clarify long-term efficacy, and evaluate cost-effectiveness across diverse healthcare settings.
